# Multiplex CRISPR/Cas9-mediated editing of seven glycosyltransferase homologs in *Nicotiana benthamiana* to produce stable, Cas9-free, glycoengineered plants

**DOI:** 10.3389/fpls.2025.1701668

**Published:** 2025-12-12

**Authors:** Chetan Kaur, Hayoung Song, Myungjin Lee, Seo-Young Kim, Dong-Hoon Seo, Hyangju Kang, Eun-Ju Sohn, Yidong Ran, Okjae Koo, Geung-Joo Lee

**Affiliations:** 1 Department of Horticulture, Chungnam National University, Daejeon, Republic of Korea; 2 Department of Smart Agriculture Systems, Chungnam National University, Daejeon, Republic of Korea; 3 BioApplications Inc., Pohang, Republic of Korea; 4 Qi Biodesign, Beijing, China; 5 Department of Veterinary Medicine, Kyungpook National University, Daegu, Republic of Korea

**Keywords:** multiplex CRISPR-Cas9 editing, homozygous gene knockouts, agrobacterium-mediated transformation, glycoengineered plants, plant molecular farming, *Nicotiana benthamiana*

## Abstract

**Introduction:**

Plant-based systems hold great potential for producing therapeutic proteins, but differences in N-glycosylation pathways between plants and mammals present major technical and regulatory barriers. In particular, plant-specific α-1,3-fucosylation and β-1,2-xylosylation can generate immunogenic glycan structures, necessitating genome engineering to humanize plant glycosylation profiles.

**Methods:**

We applied multiplex CRISPR/Cas9 genome editing in Nicotiana benthamiana to simultaneously target five α-1,3-fucosyltransferase genes and two β-1,2-xylosyltransferase genes. Resulting T_0_ transformants were genotyped to assess mutagenesis, and subsequent T1 and T2 generations were screened to identify Cas9-free, homozygous plants. Growth and morphological characteristics were evaluated across germination, flowering, and seed production stages.

**Results:**

Two T_0_ lines (HL40 and HL64) exhibited successful edits in all seven target genes, with mutations consisting of single-base insertions and deletions up to 26 bp. In later generations, we identified stable Cas9-free homozygous lines containing mutations across all targeted loci. Three T_1_ transformants with the highest number of homozygous alleles were selected to generate T_2_ progeny. Heterozygous alleles segregated into homozygous genotypes in the T_2_ generation, accompanied by confirmed loss of enzymatic activity. T_2_ plants showed no detectable morphological or growth differences compared with wild-type plants, indicating no adverse phenotypic effects. Ultimately, we generated 12 independent Cas9-free, glycoengineered, homozygous lines.

**Discussion:**

This work establishes the first *N. benthamiana* lines that are fully Cas9-free and homozygously edited at all seven key glycosyltransferase loci. These glycoengineered lines provide a stable and versatile genetic platform for future plant-based glycoengineering efforts and the production of recombinant therapeutic proteins.

## Introduction

Plant molecular farming is emerging as a leading contender for sustainable and affordable biopharmaceutical production (Yao et al., 2015; Shanmugaraj et al., 2020). Plants harness photosynthesis to generate their own energy, thereby significantly reducing overall production costs. Plant systems also exhibit a lower risk of contamination by human pathogens than animal systems. This inherent safety feature stems from the inability of human pathogens to effectively replicate within plant cells (Sil and Jha, 2014). Despite their advantages over alternative expression systems, plant-based expression systems are not without limitations. A major hurdle in plant-based protein production is the incompatibility between plant and animal protein glycosylation (Brooks et al., 2014). Glycosylation is a post-translational modification involving the enzymatic addition of sugar chains to specific amino acid residues of proteins. These covalent linkages alter protein folding, morphology, stability, and half-life, consequently influencing protein activity, signaling, and intercellular interactions (Castilho and Steinkellner, 2012; Strasser, 2016). Two types of glycosylation pathways exist: N-glycosylation and O-glycosylation. During N-glycosylation, a glycan forms a covalent bond with the nitrogen atom of an asparagine (Asn) residue. O-glycosylation, on the other hand, attaches a sugar molecule to the hydroxyl side chain of either serine (Ser) or threonine (Thr) residues. The specific pattern of N-glycosylation on a medicinal protein significantly impacts its functionality and therapeutic efficacy (Barolo et al., 2020).

Plants and animals share a conserved N-glycosylation pathway that initiates in the endoplasmic reticulum (ER). However, the later stages of N-glycan maturation differ substantially between them, as these steps are catalyzed by distinct sets of glycosyltransferases. Plant N-glycans typically contain β-1,2-xylose and α-1,3-fucose residues, whereas mammalian N-glycans possess β-mannose and α-1,6-fucose (Pattison and Amtmann, 2009). These structural differences can elicit immune responses in humans, limiting the use of plant based systems for producing therapeutic glycoproteins (Gomord et al., 2010; Rup et al., 2017). The addition of β-1,2-xylose and α-1,3-fucose residues in plants is catalyzed by β-1,2-xylosyltransferase (*XylT*) and α-1,3-fucosyltransferase (*FucT*), respectively. *Nicotiana benthamiana* contains five *XylT* and two *FucT* homologs, thus complicating complete suppression through single-gene RNAi approaches (Strasser et al., 2008). Six glycosyltransferase genes were previously targeted using CRISPR/Cas9 genome editing; however, one *FucT* homolog (*FucT5*) was omitted, and the resulting plants retained the Cas9 transgene, thus maintaining their transgenic status (Jansing et al., 2019). Cas9 free lines are required to meet biosafety and regulatory standards for the production of molecular farming crops, as transgene presence can raise concerns about off-target editing and transgene remobilization (Ahmad et al., 2024). Developing Cas9-free plants addresses both scientific and regulatory challenges. Removing Cas9 stabilizes the edits and prevents further nuclease activity. It also brings the edited plants closer to conventional breeding standards, easing regulatory evaluation and public acceptance. For these reasons, producing Cas9-free lines has become an important step toward practical CRISPR applications in agriculture. In addition to generating Cas9-free lines, it is important to fully modify the pathway to eliminate all plant specific glycosylation. Although Jansing et al. reported the absence of detectable α-1,3-fucose despite omitting *FucT5*, the functional status of this homolog remains unresolved. Given the redundancy within the *FucT* family and the polyploid genome of N. benthamiana, the possibility of residual or conditional *FucT5* activity cannot be fully ruled out.

Here, we address both limitations by targeting all seven glycosyltransferase homologs, including *FucT5*, using multiplex CRISPR/Cas9 editing to generate stable, homozygous, and Cas9 free *N. benthamiana* lines. Through genetic screening, we isolated plants carrying homozygous mutations in all target loci and confirmed complete segregation of the Cas9 transgene by the T_2_ generation. This strategy not only validates the comprehensive removal of plant-specific glycosylation but also establishes a regulatory compliant platform for scalable production of recombinant proteins with fully humanized N-glycan structures.

## Materials and methods

### Plant material and growth conditions

Forty days after germination, *Nicotiana benthamiana* seedlings were grown *in vitro* in a growth chamber under a 16-h light/8-h dark photoperiod, 140 μmol m^-2^ s^-1^ light intensity, and a temperature of 25 °C. Seeds from T_0_ transgenic plants were collected, and subsequent generations were propagated by seed harvesting and sowing. The regenerated T_1_ and T_2_ seedlings were grown in a controlled growth chamber. The chamber maintained a 16-hour light/8-hour dark photoperiod, a temperature range of 22-26°C and, a relative humidity of 40-60%.

### Cas9/sgRNA plasmid construction

Sequence information for two β-1,20-xylosyltransferase (*XylT*) genes and five α-1,3-fucosyltransferase (*FucT*) genes was obtained from the *N. benthamiana* genome data registered on the Sol Genomics Network (https://solgenomics.net). Conserved domain search was done using NCBI conserved domain database. These sequences were further compared using CLUSTAL Omega (EMBL-EBI, UK) to identify the conserved motif regions between homologous genes. gRNAs were designed using CRISPOR (http://crispor.tefor.net/; accessed March 2022) and selected based on on-target efficiency, off-target mismatch count (≥ 3 mismatches to minimize off-target cleavage < 3 %), and frame-shift probability. Each sgRNA cassette was placed downstream of the *Arabidopsis U6* promoter and assembled into the pGenovo111 binary CRISPR/Cas9 vector (Genovo Bio, Tianjin, China) using Golden Gate cloning with BsaI-HFv2 and T4 DNA ligase (37 °C 5 min / 16 °C 5 min, 25 cycles). Constructs were confirmed by Sanger sequencing and introduced into *Agrobacterium tumefaciens* strain EHA105 by electroporation. Transformation followed the Agrobacterium-mediated leaf-disc procedure: 40-day-old seedlings were inoculated with *A. tumefaciens suspension* (OD_600_ = 0.6–0.8) and co-cultivated for 3 days at 25 °C on shoot-induction medium containing 200 µM acetosyringone, 1.0 mg/L BAP, 0.1 mg/L IAA, and 0.3 % Gelrite. Shoots were selected on MS medium with 20 µg/mL hygromycin and 150 mg/L Timentin, elongated on 0.3 mg/L BAP + 0.1 mg/L IAA, and rooted on ½ MS medium with 15 µg/mL hygromycin. Regenerated plants were transferred to soil and grown under 16 h light/8 h dark at 20–24 °C and 40–60 % RH. Seeds were obtained by self pollination for segregation and homozygosity analysis Song et al., 2022).

### DNA isolation and analysis of gene modifications

The leaves of the regenerated plants were used to extract the total genomic DNA using a Deasy Plant Mini Kit (QIAGEN, Hilden, Germany). Fragments spanning each of the seven target loci (*NbFucT1–5*, *NbXylT1*, *NbXylT2*) were amplified by PCR using gene-specific primers (**Supplementary Table S1**). PCR products were analyzed for mutations using Sanger sequencing first, followed by targeted next-generation sequencing (NGS) for more comprehensive coverage. For NGS, three successive high fidelity PCR amplifications were performed to enrich the target region while minimizing amplification bias. Each 25 µL reaction contained 50 ng genomic DNA, 0.5 µM primers, 200 µM dNTPs, 1× Phusion HF buffer, and 0.5 U Phusion High-Fidelity DNA Polymerase (Thermo <ns/>F530L). The second and third PCR reactions used 1/10 and 1/100 dilutions of the previous reaction as templates, without intermediate purification. Amplicons (~350 bp) were pooled, purified with AMPure XP beads (Beckman Coulter), and dual indexed libraries were prepared using the NEBNext Ultra II DNA Library Prep Kit (New England Biolabs). Libraries were sequenced on an Illumina MiSeq platform. Raw reads were demultiplexed and quality-trimmed (Phred > 30) using Trimmomatic v0.39, and low-quality reads were discarded. Clean reads were aligned to the *N. benthamiana* reference genome and analyzed using the CRISPR RGEN Cas-Analyzer pipeline to quantify insertions, deletions, and single-nucleotide variants within ± 10 bp of each Cas9 cleavage site. Mutations were classified as homozygous when identical alleles accounted for ≥ 80 % of reads and as bi-allelic when two distinct alleles each exceeded 30 % frequency.

### Total protein N-glycan analysis by Western blot

Total soluble protein was extracted from 100 mg of *N. benthamiana* leaf tissue by grinding in two volumes of ice cold PBS (137 mM NaCl, 2.7 mM KCl, 8.1 mM Na_2_HPO_4_, 1.5 mM KH_2_PO_4_, pH 7.4). The homogenate was centrifuged at 16,100 × g for 10 min at 4 °C, and 10 µg of total protein per lane was resolved by SDS–PAGE (100 V, 90 min) and transferred to a nitrocellulose membrane (Thermo Fisher Scientific, MA, USA) using Towbin transfer buffer (25 mM Tris, 192 mM glycine, 20% methanol) for 1 h at 100 V, following the general workflow of Jansing et al (Jansing et al., 2019). Membranes were blocked for 1 h in 5% skim milk in TBS–T (20 mM Tris, 150 mM NaCl, 0.05% Tween-20, pH 7.5) and briefly rinsed. Blots were incubated with primary antibodies from Agrisera AB (Umeå, Sweden): anti-β-1,2-xylose (AS07 267, 1:5000) or anti-α-1,3-fucose (AS07 268, 1:10000), diluted in standard or high-salt TBS-T (20 mM Tris, 500 mM NaCl, 0.1% Tween-20), respectively. The two glycan specific blots were performed on separate membranes. Following primary incubation, membranes were washed three times (5 min each) in the corresponding TBS-T buffer and probed for 1 h with alkaline-phosphatase-conjugated goat anti-rabbit IgG (H + L) secondary antibody (AS09 607, Agrisera, 1:10000), pre-adsorbed against wild-type *N. benthamiana* extract to reduce non-specific binding. After three final washes, membranes were developed in BCIP/NBT substrate (Thermo Fisher Scientific) for 5–15 min in the dark, rinsed in distilled water, and air dried.

### Validation of Cas9 integration

The presence of Cas9 within the transformants was verified using Cas9 gene-specific genomic PCR. Each 20 µL reaction contained 1 µL genomic DNA (30–50 ng), 1 µL each of Cas9 forward and reverse primers (10 pmol/µL), 1 µL each of *NbActin* control primers (10 pmol/µL), 10 µL of 2× PCR premix (Takara Ex Taq, Japan), and 7 µL nuclease-free water. PCR was performed with an initial denaturation at 94°C for 5 min; 32 cycles of 94°C for 30 s, 58°C for 30 s, and 72°C for 30 s; followed by a final extension at 72°C for 7 min. PCR products were visualized using a gel documentation system under UV light. The expected product sizes were 1448 bp for *Cas9* and 267 bp for *NbActin* (primer sequences listed in **Supplementary Table S1**).

## Results

### Design and assembly of Cas9/sgRNA constructs for generating multiple knockout transformants

**Figure 1:**
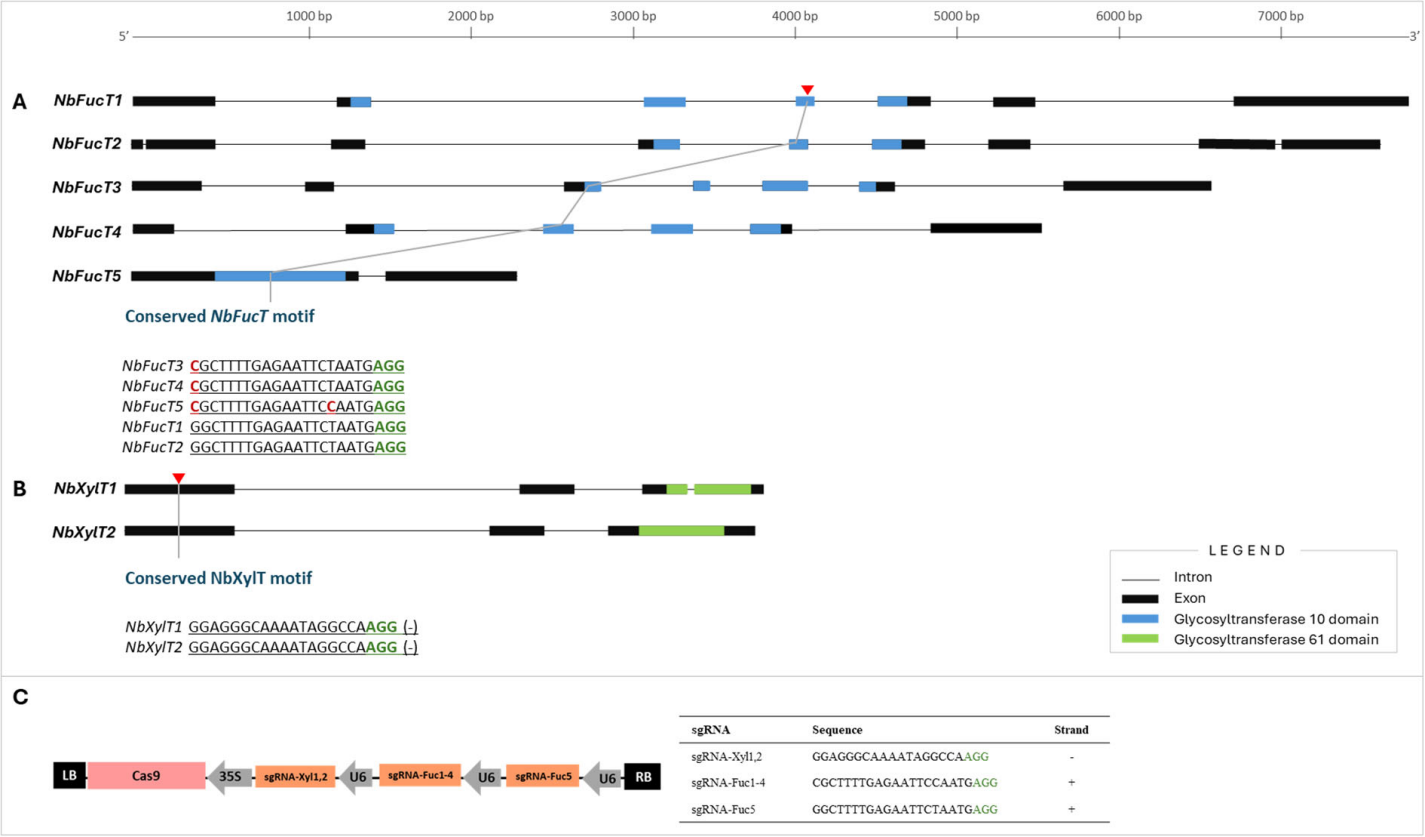
Selection of target sites for multiplexed genome editing to knockout in five *NbFucT* and two *NbXylT* genes using CRISPR-Cas9. Target sites in **(A)***NbFucT* and **(B)***NbXylT* genes. **(C)** Schematic diagram of the Cas9-sgRNA construct. The black boxes indicate exons, and the underlined sequences correspond to the sgRNA. The red and green nucleotides represent SNPs and PAM sequences, respectively. .

Sequence information of the two *XylT* genes (Niben101Scf04551:*NbXylT1*, Niben101Scf04205: *NbXylT2*) and five *FucT* genes (Niben101Scf01272:*NbFucT1*, Niben101Scf02631:*NbFucT2*, Niben101Scf05494: *NbFucT3*, Niben101Scf17626: *NbFucT4*, Niben101Scf05447: *NbFucT5)* present in *N. benthamiana* were aligned and analyzed to identify conserved motifs and domains (**Figures 1A, B**). The *XylT* genes consisted of a conserved domain, belonging to the Glycosyltransferase 61 domain family, and the *FucT* genes consisted of a conserved domain belonging to the Glycosyltransferase 10 domain family. Notably, PFAM/HMMER analysis identified this canonical GT10 domain in *FucT5* (E = 2.16 × 10^-^²^9^), identical to the catalytic domain found in *FucT1–4*. Reciprocal BLASTP comparisons further revealed high sequence identity and similarity among *FucT1–5*. These results indicate that *FucT5* retains the conserved catalytic core characteristic of functional α-1,3-fucosyltransferases. Based on the conserved motifs of each gene, three sgRNAs were designed to simultaneously target seven genes: sgRNA-Xyl1,2 targets *NbXylT1* and *NbXylT2*, and sgRNA-Fuc1–4 targets *NbXylT1*, *NbXylT2*, *NbXylT3*, and *NbXylT4* (**Figure 1**). Additionally, sgRNA-Fuc5 was designed to target *NbFucT5* to increase the target specificity due to a polymorphism in the 5th sequence from the PAM site. As for *NbXylTs*, reverse sgRNAs were designed by referring to the 100%-matching conserved sequences identified on exon 1 of *NbXylT1* and *NbXylT2* (**Figure 1B**). Three gRNAs targeting the five *NbFucT* and two *NbXylT* genes were inserted into the Cas9-containing plasmid (**Figure 1C**). Agrobacterium-mediated transformation was used to deliver the designed Cas9 containing DNA constructs (pk-NbFUCT-XYLT) into plant cells, resulting in a successful generation of transformants.

### Evaluation and characterization of the mutations induced by multiplexed Cas9 targeting of the *XylT* and the *FucT* genes

**Figure 2:**
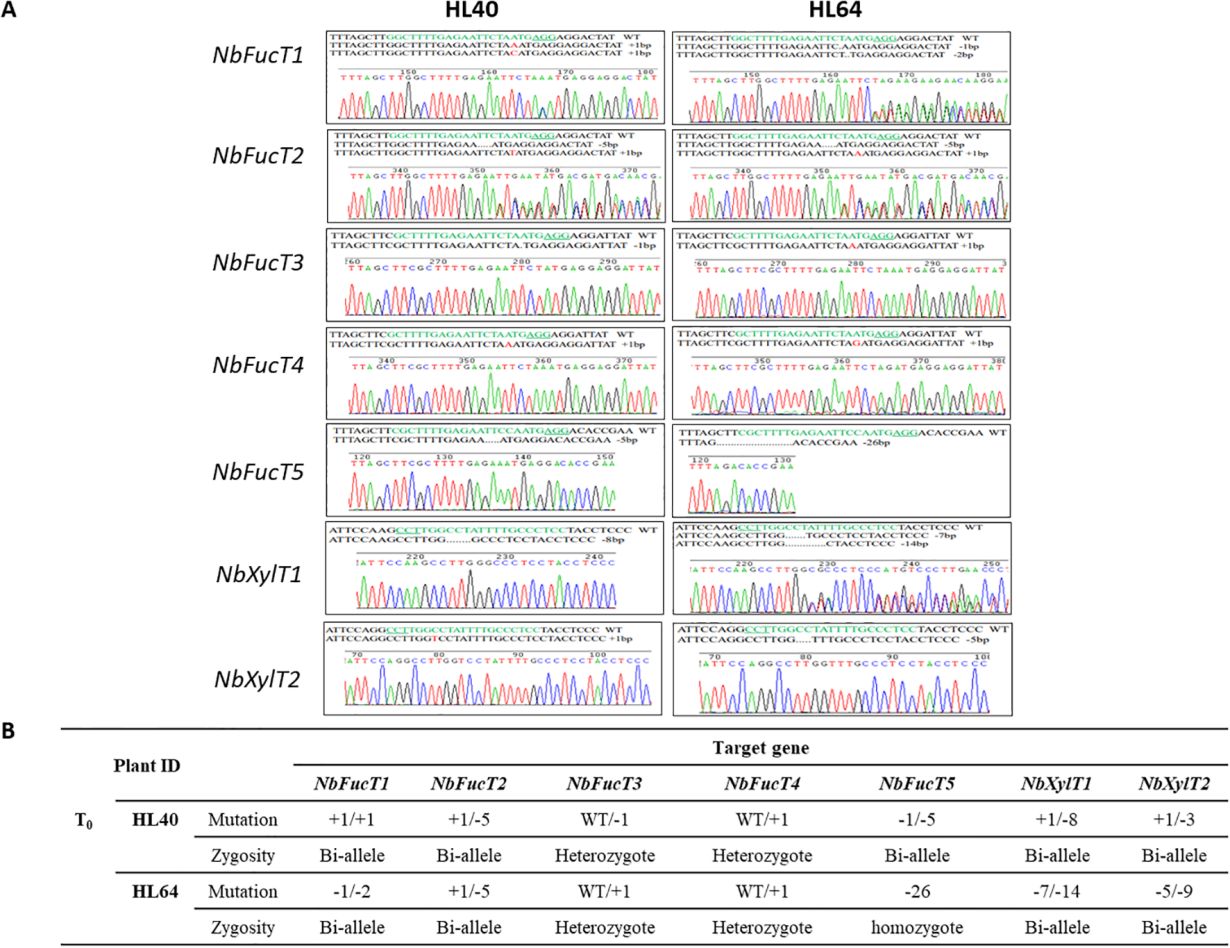
Mutations induced in *N. benthamiana* by the CRISPR/Cas9 system. **(A)** Results of mutation analysis of 7 target sites identified in HL40 and HL60 T_0_ transgenic plants. **(B)** Genotype and zygosity analysis of T_0_ transformants.

Mutations resulting from the editing of the five *NbFucT* and two *NbXylT* genes were identified by Sanger sequencing of the target gene fragment (**Figure 2A**). Among these transformants, two plant lines, HL40 and HL64, were selected, each of which exhibited mutations in all seven genes (**Table 1**). The transformant line HL40 exhibited bi-allelic mutations at five target gene sites which included one base insertion in *NbFucT1*, one base insertion or five base deletions in *NbFucT2*, one base deletion or five base deletions in *NbFucT5*, one base insertion or eight base deletions in *NbXylT1*, and one base insertion or three base deletions in *NbXylT2* (**Figure 2B**). Heterozygous mutations were identified in the target regions on *NbFucT3* and *NbFucT4* with one base deletion and one base insertion, respectively. In the transformant line HL64, bi-allelic mutations were identified at four target sites: one- or two-base deletions in *NbFucT1*, one- or five-base deletions in *NbFucT2*, seven- or fourteen-base deletions in *NbXylT1*, and five- or nine-base deletions in *NbXylT2.* The target sites *NbFucT3* and *NbFucT4* in HL64 exhibited heterozygous mutations with one base insertion each. Additionally, a deletion of 26 bases was observed in the HL64 *NbFucT5* target site (**Figure 2B**).

**Table 1:** Characterization of mutations in the target genes of the T2 transformant plant lines. (*Cas9 free plants).

CRISPR/Cas9-induced mutations											
Generation	T0	T1	T2		NbFUCT1	NbFUCT2	NbFUCT3	NbFUCT4	NbFUCT5	NbXYLT1	NbXYLT2
Plant IDs	HL40			Mutation	+1(A)/+1(C)	+1/-5	WT/-1	WT/+1	-1/-5	+1/-8	+1/-3
				Zygosity	Bi-allele	Bi-allele	Heterozygote	Heterozygote	Bi-allele	Bi-allele	Bi-allele
		HL40-48*		Mutation	+1bp(A)	-+1/-5	-1	+1	-5	-8	+1
				Zygosity	Homozygote	Bi-allele	Homozygote	Homozygote	Homozygote	Homozygote	Homozygote
			HL40-48-1*	Mutation	+1bp(A)	-5bp	-1bp	+1bp	-5bp	-8bp	+1bp
				Zygosity	Homozygote	Homozygote	Homozygote	Homozygote	Homozygote	Homozygote	Homozygote
			HL40-48-2*	Mutation	+1bp(A)	-5bp	-1bp	+1bp	-5bp	-8bp	+1bp
				Zygosity	Homozygote	Homozygote	Homozygote	Homozygote	Homozygote	Homozygote	Homozygote
			HL40-48-3*	Mutation	+1bp(A)	+1bp	-1bp	+1bp	-5bp	-8bp	+1bp
				Zygosity	Homozygote	Homozygote	Homozygote	Homozygote	Homozygote	Homozygote	Homozygote
			HL40-48-4*	Mutation	+1bp(A)	+1bp/-5bp	-1bp	+1bp	-5bp	-8bp	+1bp
				Zygosity	Homozygote	Bi-allele	Homozygote	Homozygote	Homozygote	Bi-allele	Homozygote
		HL40-219*		Mutation	+1bp(C)	-5bp	-1bp	+1bp	-1bp/-5bp	+1bp	+1bp
				Zygosity	Homozygote	Homozygote	Homozygote	Homozygote	Bi-allele	Homozygote	Homozygote
			HL40-219-1*	Mutation	+1bp(C)	-5bp	-1bp	+1bp	-1bp/-5bp	+1bp	+1bp
				Zygosity	Homozygote	Homozygote	Homozygote	Homozygote	Bi-allele	Homozygote	Homozygote
			HL40-219-2*	Mutation	+1bp(C)	-5bp	-1bp	+1bp	-1bp/-5bp	+1bp	+1bp
				Zygosity	Homozygote	Homozygote	Homozygote	Homozygote	Bi-allele	Homozygote	Homozygote
			HL40-219-3*	Mutation	+1bp(C)	-5bp	-1bp	+1bp	-5bp	+1bp	+1bp
				Zygosity	Homozygote	Homozygote	Homozygote	Homozygote	Homozygote	Homozygote	Homozygote
			HL40-219-4*	Mutation	+1bp(C)	-5bp	-1bp	+1bp	-1bp/-5bp	+1bp	+1bp
				Zygosity	Homozygote	Homozygote	Homozygote	Homozygote	Bi-allele	Homozygote	Homozygote
	HL64			Mutation	-1bp/-2bp	+1bp/-5bp	WT/+1bp	WT/+1bp	-26bp	-7bp/-14bp	-5bp/-9bp
				Zygosity	Bi-allele	Bi-allele	Heterozygote	Heterozygote	Homozygote	Bi-allele	Bi-allele
		HL64-512*		Mutation	-1/-2	+1	+1	+1	-26	-7/-14	-5
				Zygosity	Bi-allele	Homozygote	Homozygote	Homozygote	Homozygote	Bi-allele	Homozygote
			HL64-512-1*	Mutation	-2bp	+1bp	+1bp	+1bp	-26bp	-7bp	-5bp
				Zygosity	Homozygote	Homozygote	Homozygote	Homozygote	Homozygote	Homozygote	Homozygote
			HL64-512-2*	Mutation	-1bp/-2bp	+1bp	+1bp	+1bp	–26bp	-7bp	-5bp
				Zygosity	Bi-allele	Homozygote	Homozygote	Homozygote	Homozygote	Homozygote	Homozygote
			HL64-512-3*	Mutation	-1bp	+1bp	+1bp	+1bp	-26bp	-7bp	-5bp
				Zygosity	Homozygote	Homozygote	Homozygote	Homozygote	Homozygote	Homozygote	Homozygote
			HL64-512-4*	Mutation	-1bp/-2bp	+1bp	+1bp	+1bp	-26bp	-7bp-14bp	-5bp
				Zygosity	Bi-allele	Homozygote	Homozygote	Homozygote	Homozygote	Bi-allele	Homozygote

* Cas-9 Free Plants.

### Identification of T_1_*FucT* and *XylT* knockout lines

T_0_ plants of lines HL40 and HL64 were self-pollinated to produce T_1_ generation plants. Two screening strategies were applied to identify transformants among T_1_ generation plants: PCR analysis to select lines free of the Cas9 transgene, and Western blotting with fucose and xylose antibodies to confirm the absence of α-1,3-fucose and β-1,2-xylose residues, indicating targeted gene silencing. Western blot analysis of the T_1_ transformants revealed no fucose residues in line HL40–4 and no xylose residues in lines HL40-11, HL40-14, HL40-18, HL40-20, HL64-15, and HL64-17 (**Supplementary Figure S2**). In HL64-19, neither fucose nor xylose residues were detected (**Supplementary Figure S2B**). Eight T_1_ transformants displaying loss-of-function of the target gene were selected for sequencing.

Additional screening of the Cas9-free T_1_ transformants lacking the target genes was performed using primers specifically designed to amplify the Cas9 and sgRNA fragments within the engineered plasmid. (**Supplementary Table S1**, **Supplementary Figure S3**). Five T_1_ lines lacking the amplification products for both sets of primers were selected: HL40-48, HL40-219, HL40-379, HL-40-591, and HL64-512 (**Supplementary Figure S3**). As a result of these two screening processes, 13 T_1_ transformants were selected for further analysis of the genotypes and segregation patterns of the mutations using targeted deep sequencing (**Supplementary Table S2**).

The T_1_ transformant HL40-4, which lacked α-1,3-fucose residues, inherited the same heterozygous mutations from the T_0_-HL40 parent, in the target sites *NbFucT5*, *NbXylT1*, *NbXylT2*, whereas all the other target genes inherited the mutation in a homozygous pattern. Another T_1_ transformant lacking the α-1,3-fucose residues; HL64-19, inherited the heterozygous mutation pattern from the T_0_-HL64 parent in the target sites NbFucT1, *NbXylT1*. All the other target genes showed inheritance of the mutation in a homozygous pattern (**Supplementary Table S2**). Sequencing of the T_1_ transformants HL40-11, HL40-14, HL40-18, and HL40–20 revealed the presence of a wild-type allele in the target regions of *NbFucT3* and *NbFucT4*. Western blot analysis revealed the absence of β1,2-xylose residues but the presence of α-1,3-fucose residues in these transformants. This suggests that a complete knockout of the α-1,3-fucose phenotype, as evidenced by the lack of fucose residues, likely requires the absence of the wild-type allele in both *NbFucT3* and *NbFucT4* genes. In HL40-4, β1,2-xylose residues were detected despite the absence of the wild-type allele in either of the *NbXylT* target genes. In contrast, HL40-20, which lacked β1,2-xylose residues, shared the same heterologous mutation at the *NbXylT1* target site as HL40–4 but inherited only one base homologous insertion at the *NbXylT2* target site. Consequently, it can be inferred that the three-base deletion in the *NbXylT2* site did not significantly affect the β-1,2-xylosyltransferase activity. All lines selected through PCR screening (HL40-48, HL40-219, HL40-379, HL-40-591, and HL64-512) lacked the wild-type allele, whereby it could be anticipated that the mutation pattern in these lines would result in loss of function of all *NbFucT* and *NbXylT* target genes.

### Generation of Cas9-free, stable FucT and XylT knockout T_2_ plants

**Figure 3:**
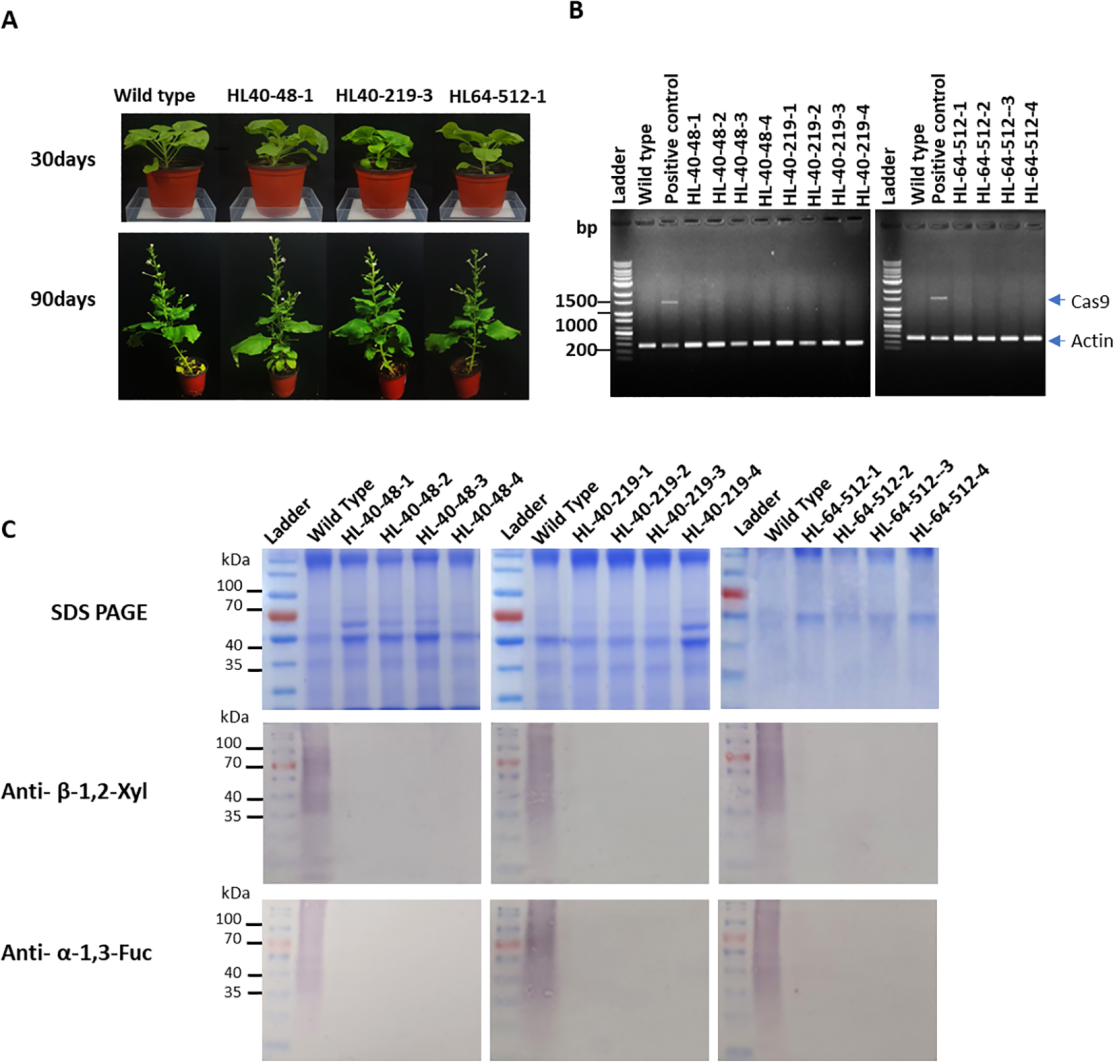
Phenotypic comparison and molecular characterization of transformants. **(A)** Morphological comparison of T_2_ generation plants throughout their growth stages. Plants were photographed 30 and 90 days after sowing. **(B)** PCR-based reconfirmation of absence of Cas9 in T_2_ transformants. Wild type *N. benthamiana* was used as negative control. HL-64–19 T_1_ plant was used as positive control. HL40-48-1~4, HL40-219-1~4, HL64-512-1~4 T2 plants were Cas9-free. Actin amplification product size: 267 bp, Cas9 amplification product size:1448 bp. **(C)** Western blot analysis for comparison of α-1,3-fucose and β-1,2-xylose residues in *NbFucT* and *NbXylT* transformants and wild-type. Wild-type tobacco was used as positive control. Results of anti- β-1,2-XylT is β-1,2-xylosyltransferase antibody reaction results.

Of the five Cas9-free T_1_ plants, three T_1_ transformants (HL40-48, HL40-219, and HL64-512) were selected for the production of T_2_ plants because these lines showed the highest number of homozygous mutations. In particular, these selected transformants, HL40-48, HL40-219, and HL64-512, showed six homozygous mutations and one heterozygous mutation in NbFucT2, NbFucT5, and NbXylT2, respectively. To obtain stable homozygous mutant lines for F-KO and X-KO, individuals from these lines were cultivated and sequencing of the target sites was performed. The morphology and growth rate of the T_2_ plants were also observed to detect the side effects of the mutations. No significant differences were observed between T_0_ and T_2_ generation plants compared to wild-type plants in terms of external morphological characteristics, from germination to flowering, and seed production (**Figure 3A**). Sequence analysis revealed the segregation of heterozygous alleles into homozygous genotypes in the T_2_ generation. Among the T_2_ plants derived from the HL-40–48 T_1_ line, the plants identified as HL40-48-1, HL 40-48-2, and HL40-48–3 exhibited homozygous genotypes in all seven target regions (**Table 1**). Similarly, T_2_ plants HL40-219-3, HL64-512-1, and HL64-512–3 were confirmed to have homozygous mutations in the seven target genes (**Table 1**). Furthermore, T_2_ plants HL40-48-1~4, HL40-219-1~4, and HL64-512-1~4 were all Cas9-free (**Figure 3B**).

Western blot analysis on selected T2 lines provided final confirmation of functional enzyme loss for all seven targeted genes. This analysis revealed the absence of both β1,2-xylose and α-1,3-fucose residues in the T2 transformants, solidifying the successful knockout of all seven target genes (**Figure 3C**).

## Discussion

Harnessing plants as factories for therapeutic proteins holds immense promise for enhancing and optimizing the synthesis of proteins that are crucial for treating and preventing diseases in humans and animals. There are significant advantages to using this strategy; however, successful implementation requires addressing technical and regulatory hurdles. Although several vaccines, antibiotics, and therapeutic proteins have been developed in plants (Obembe et al., 2011), the generation of mutants lacking the activity of key enzymes (e.g., β-1,2-xylosyltransferase and α-1,3-fucosyltransferase) prevents the formation of certain epitopes on plant N-glycans. The effectiveness of this approach was validated by generating *Arabidopsis thaliana* knockout mutants, including a triple knockout line that displayed normal plant development under standard growth conditions (Strasser et al., 2004).

*N. benthamiana* has shown promise as a potential host for producing recombinant glycoproteins with customized N- and O-glycan modifications (Strasser et al., 2008; Jansing et al., 2019). Random mutagenesis studies using EMS demonstrated that a 4- or 5-fold knockout of exclusively the fucosyltransferase genes resulted in a decrease in fucose levels but unexpectedly led to an increase in xylose levels in plants (Nagels et al., 2011). This indicates a compensatory mechanism in glycan synthesis, where the absence of fucose may trigger an upregulation of xylose incorporation. A complete disruption of both fucose and xylose biosynthetic pathways is necessary to significantly alter the glycan profile. Therefore, a 7-fold knockout of fucosyltransferase and xylosyltransferase genes is more effective in disrupting glycan synthesis than a 4- or 5-fold knockout. This is likely due to the elimination of redundant pathways, the suppression of compensatory mechanisms, and the synergistic effect of targeting both types of genes, resulting in a greater reduction of fucose and xylose levels in the plant. The advent of techniques like multiplex gene editing, base editing, prime editing and Cas12a/CPpf1 have accelerated the progress in plant glycoengineering Abdelrahman et al., 2021). Recent advances in gene editing, including base editing, prime editing and multiplex CRISPR strategies, have expanded the toolkit for precise genome manipulation in plants. Base editing and prime editing offer powerful options for targeted nucleotide substitutions, small insertions or corrections, and motif-level refinements without introducing double-strand breaks (Molla et al., 2021). These tools are especially useful for fine tuning enzyme function, modifying catalytic residues and generating allelic variants with subtle but biologically meaningful changes. However, their current delivery limitations, lower efficiency in several crop species and complexities in multiplexing make them less suited for large-scale gene family knockouts. Multiplex CRISPR/Cas9 gene editing remains the most efficient and practical strategy for simultaneously disrupting several homologous genes to completely abolish their function, especially in systems in which multiple paralogs act redundantly within the same biochemical pathway.

**Figure 4:**
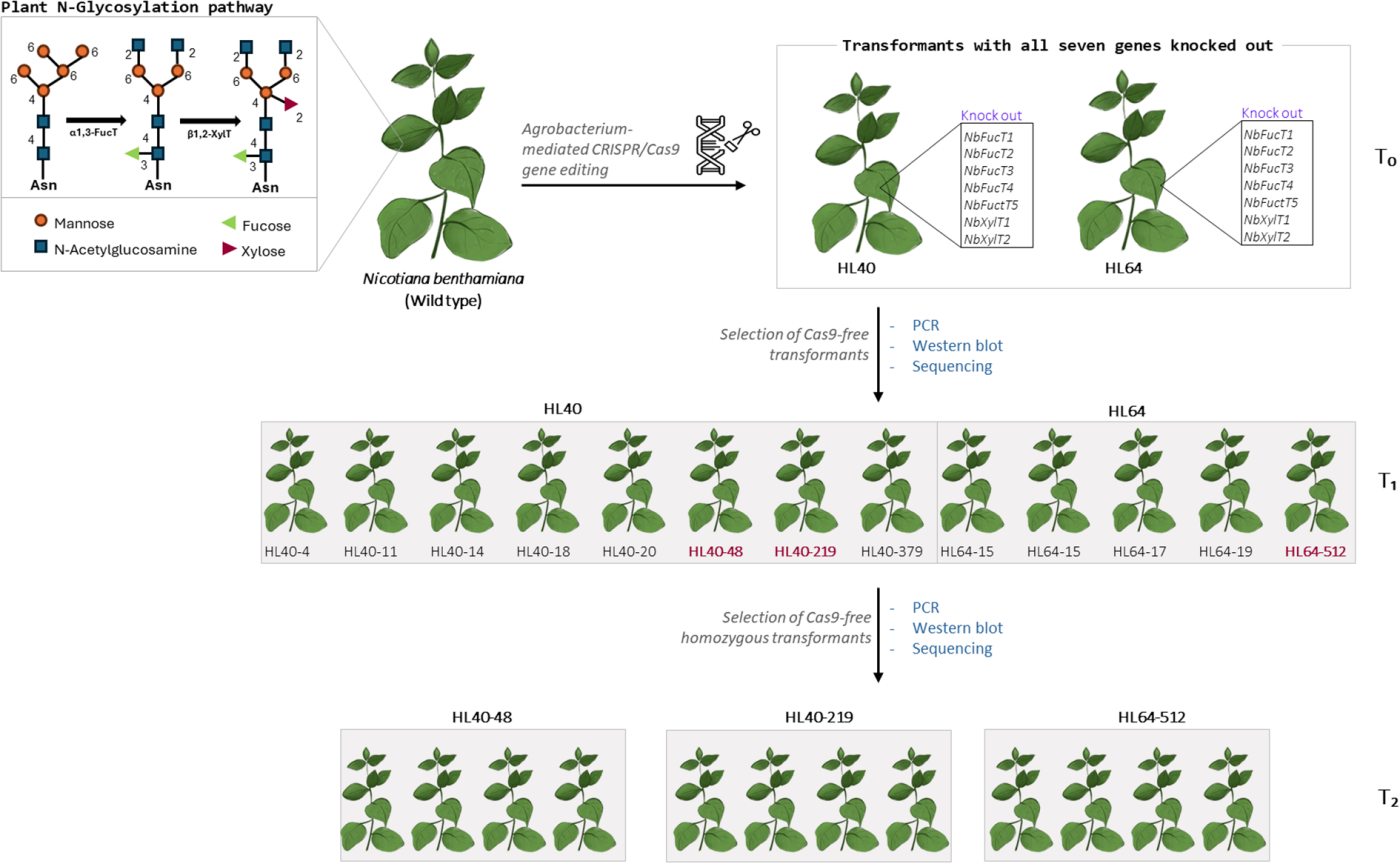
Summary of CRISPR/Cas9 mediated knockout of seven N-glycosylation genes in *Nicotiana benthamiana* and selection of Cas9-free homozygous transformants in the consecutive generations (T1 and T2).

Building on our previous work targeting *NbXylT1* and *NbFucT2* to characterize mutation nature and inheritance, we expanded our approach to achieve near complete elimination of *FucT* and *XylT* activity by simultaneously targeting all seven genes Song et al., 2022). This represents, to our knowledge, the first successful multiplex CRISPR/Cas9 editing of all seven glycosyltransferase homologs in *N. benthamiana*, addressing limitations of previous studies that excluded the *FucT5* homolog. We employed Agrobacterium-mediated CRISPR/Cas9 to achieve targeted editing of all homologous *NbXylT* and *NbFucT* genes. A systematic analysis of mutation rates, types, and genetic trends was performed on the transformed sample. Two T0 transformants were identified that successfully carried mutations in all seven target genes, HL40 and HL64. Analysis of the mutational characteristics in the selected T0 mutants revealed deletions and insertions as the most frequent types of induced edits. Notably, insertions were predominantly single nucleotide additions, while deletions spanned a wider range, from one to twenty-six nucleotides. The *NbFucT3* and *NbFucT4* genes showed heterozygous mutations carrying the wild-type allele in both the HL40 and HL64 transformants, whereas the other target gene mutations were bi-allelic or homozygous. Despite the expected segregation of alleles, a few plants in the T1 generation retained the wild-type allele, which has been the case for many other plant edits using CRISPR/Cas9 (Pan et al., 2016; Cheng et al., 2019). From the three T1-Cas9 free plants, which carried homozygous mutations in all but one target gene site, we generated T2 transformant lines that carried a stable mutation in all seven target genes and were Cas-9 free (**Figure 3**). Loss of enzymatic activity for both *NbFucT* and *NbXylT* was confirmed in all T2 transformants. The external morphology and growth of T2 plants remained largely similar to those of T0 and wild-type plants throughout development, providing preliminary evidence of minimal side effects induced by the mutations (**Figure 3A**). The lack of observable morphological or growth defects in T2 plants suggests these multiplex gene edits do not impair plant viability or development, which is essential for their use as production platforms. Overall, this study provides a solid genetic platform for glycoengineering in *N. benthamiana*, overcoming prior limitations by achieving full knockout of all seven glycosyltransferase homologs in stable, Cas9-free lines. In addition to achieving Cas9-free edited lines, our approach ensures complete pathway coverage by including *FucT5*, which was previously omitted. Sequence and domain analyses confirm that *FucT5* contains the GT10 catalytic core and shares high sequence similarity with other *NbFucT* homologs, supporting its potential functionality. This comprehensive targeting strategy prevents residual or context-dependent α-1,3-fucosylation and provides a more reliable foundation for downstream biopharmaceutical protein production. The study has been graphically summarized in **Figure 4**.

## Data Availability

The datasets presented in this study can be found in online repositories. The names of the repository/repositories and accession number(s) can be found in the article/**Supplementary Material**.
