# Supplementary material for: Multiplex CRISPR/Cas9-mediated editing of seven glycosyltransferase homologs in Nicotiana benthamiana to produce stable, Cas9-free, glycoengineered plants
Source: Front Plant Sci. 2025 Dec 12;16:1701668. doi: 10.3389/fpls.2025.1701668 (PMC12741666; doi:10.3389/fpls.2025.1701668)
Supplement: Supplementary file 1 [file DataSheet1.docx]

**Supplementary tables and figures**

**Table S1**. List of primers used in the study.

| **Purpose** |  | **Primer name** |  | **Primer sequence (5'-3')** |
| --- | --- | --- | --- | --- |
| **Targeted deep sequencing** | 1^st^ PCR | NbXylT1 | XYLT1 MS F1 | ACGTTGTTTAACCCTCTGTAGTCTAC |
|  |  |  | XYLT1 MS R1 | GGAAAGCTCCATTTTTGAACATG |
|  |  | NbXylT2 | XYLT2 MS F1 | TCCCACCCTGATCACTCTC |
|  |  |  | XYLT2 MS R1 | GAAAAGCTCCAGTTTTGAACGCG |
|  |  | NbFucT1 | FUCT1 MS F1 | TTAGTGATTCGAATTTATGCTAGTCTT |
|  |  |  | FUCT1 MS R1 | GTCTAGATTCAAACCATTGTTGGA |
|  |  | NbFucT2 | FUCT2 MS F1 | GTGATTCGAATTTATGCTAGTCCAATGAT |
|  |  |  | FUCT2 MS R1 | GTCTAGATCCAAACCATCGTAGAA |
|  |  | NbFucT3 | FUCT3 MS F1 | GTAGAATATGCATGCATGTGGTTAAAC |
|  |  |  | FUCT3 MS R1 | GAGCAGAGCATATTTGTGAACC |
|  |  | NbFucT4 | FUCT4 MS F1 | GTAGAATATGCATGCCTGTCGTTTGAC |
|  |  |  | FUCT4 MS R1 | TAGAGCAGAGCATATTTGTACGTCG |
|  |  | NbFucT5 | FUCT5 MS F1 | GTTGATCGGAATTTGGCAACT |
|  |  |  | FUCT5 MS R1 | CTCCAATGACAAATTAGATGACCTTAG |
|  | 2^nd^ PCR | NnFucT1&2 | NbFUT 1&2 F2 | ACACTCTTTCCCTACACGACGCTCTTCCGATCTTGGTTTCTGTTCTCAATTTG |
|  |  |  | NbFUT 1&2 R2 | GTGACTGGAGTTCAGACGTGTGCTCTTCCGATCTCAATTCTGAAAATCCAAGTC |
|  |  | NbFucT3 or 4 | NbFUT 3&4 F2 | ACACTCTTTCCCTACACGACGCTCTTCCGATCTCTTGTCATCTTGTTCTCAA |
|  |  |  | NbFUT 3 R2 | GTGACTGGAGTTCAGACGTGTGCTCTTCCGATCTAGTGTAAAGAGCAGAACC |
|  |  |  | NbFUT 4 R2 | GTGACTGGAGTTCAGACGTGTGCTCTTCCGATCTAGTGTAAAATGCAGAACC |
|  |  | NbFucT5 | NbFUT 5 F2 | ACACTCTTTCCCTACACGACGCTCTTCCGATCTCTAATTGTGGTGCTCGTAA |
|  |  |  | NbFUT 5 R2 | GTGACTGGAGTTCAGACGTGTGCTCTTCCGATCTCAGCTACTAAAGACTGGAA |
|  |  |  | NbFUT 5 F2 -2 | ACACTCTTTCCCTACACGACGCTCTTCCGATCTCTTCCGGTTGCAAGCTCTTGAA |
|  |  |  | NbFUT 5 R2-2 | GTGACTGGAGTTCAGACGTGTGCTCTTCCGATCTAGCACCAATCACCACGGGGACT |
|  |  | NbXylT1&2 | NbXYylT 1&2 F2 | ACACTCTTTCCCTACACGACGCTCTTCCGATCTCAACTCAATCACTCTCTATC |
|  |  |  | NbXylT 1&2 R2 | GTGACTGGAGTTCAGACGTGTGCTCTTCCGATCTATTACCGAAGTAACCCT |
| **Transgene-free, Cas9 free selection** |  | NF1 |  | TGTCCCAGGATTAGAATGATTAGGC |
|  |  | X6 R |  | GACGTAAGGGATGACGCACA |
|  |  | Cas9-F1 |  | GTTCATCAAGCCGATTCTGG |
|  |  | Cas9-R1 |  | GCTTCCTGCTCAGCCTCCC |
|  |  | Cas9-F2 |  | CCCACCATCTACCATCTGCG |
|  |  | Cas9-R |  | ATGTCCTCGTTCTCCTCGTTGT |

**Table S2.** Inheritance of targeted mutations and Cas9 in T_1_ progeny of transformant

| **Mutant plants** | |  | **CRISPR/Cas9-induced mutations** | | | | | | |
| --- | --- | --- | --- | --- | --- | --- | --- | --- | --- |
| **Generation** | **Plant ID** |  | **NbFUCT1** | **NbFUCT2** | **NbFUCT3** | **NbFUCT4** | **NbFUCT5** | **NbXYLT1** | **NbXYLT2** |
| **T_0_** | **HL40** | Mutation | +1/+1 | +1/-5 | WT/-1 | WT/+1 | -1/-5 | +1/-8 | +1/-3 |
|  |  | Zygosity | Bi-allele | Bi-allele | Heterozygote | Heterozygote | Bi-allele | Bi-allele | Bi-allele |
| **T_1_** | **HL40-4** | Mutation | +1 | +1 | -1 | +1 | -1/-5 | +1/-8 | +1/-3 |
|  |  | Zygosity | Homozygote | Homozygote | Homozygote | Homozygote | Bi-allele | Bi-allele | Bi-allele |
|  | **HL40-11** | Mutation | +1/+1 | +1/-5 | WT/-1 | WT/chi | -1/-5 | +1/-8 | +1 |
|  |  | Zygosity | Bi-allele | Bi-allele | Heterozygote | Chimera | Bi-allele | Bi-allele | Homozygote |
|  | **HL40-14** | Mutation | +1/+1 | +1 | WT/-1 | WT/chi | -5 | -8 | +1 |
|  |  | Zygosity | Bi-allele | Homozygote | Heterozygote | Chimera | Homozygote | Homozygote | Homozygote |
|  | **HL40-18** | Mutation | +1 | +1/-5 | WT/chi | WT/+1 | -1 | +1/-8 | +1 |
|  |  | Zygosity | Homozygote | Bi-allele | Chimera | Heterozygote | Homozygote | Bi-allele | Homozygote |
|  | **HL40-20*** | Mutation | +1 | +1 | WT | +1 | -1 | +1/-8 | +1 |
|  |  | Zygosity | Homozygote | Homozygote | Wild | Homozygote | Homozygote | Bi-allele | Homozygote |
|  | **HL40-48*** | Mutation | +1 | -+1/-5 | -1 | +1 | -5 | -8 | +1 |
|  |  | Zygosity | Homozygote | Bi-allele | Homozygote | Homozygote | Homozygote | Homozygote | Homozygote |
|  | **HL40-219*** | Mutation | +1 | -5 | -1 | +1 | -1/-5 | +1 | +1 |
|  |  | Zygosity | Homozygote | Homozygote | Homozygote | Homozygote | Bi-allele | Homozygote | Homozygote |
|  | **HL40-379*** | Mutation | +1/+1 | +1/-5 | -1 | +1 | -5 | -8 | +1 |
|  |  | Zygosity | Bi-allele | Bi-allele | Homozygote | Homozygote | Homozygote | Homozygote | Homozygote |
|  | **HL40-591*** | Mutation | +1/+1 | +1/-5 | -1 | +1 | -1/-5 | +1/-8 | +1 |
|  |  | Zygosity | Bi-allele | Bi-allele | Homozygote | Homozygote | Bi-allele | Bi-allele | Homozygote |
| **T_0_** | **HL64** | Mutation | -1/-2 | +1/-5 | WT/+1 | WT/+1 | -26 | -7/-14 | -5/-9 |
|  |  | Zygosity | Bi-allele | Bi-allele | Heterozygote | Heterozygote | Homozygote | Bi-allele | Bi-allele |
| **T_1_** | **HL64-15** | Mutation | -1/-2 | -5 | WT | WT/+1 | -26/Chi | -7/-14 | -5 |
|  |  | Zygosity | Bi-allele | Homozygote | Wild | Heterozygote | Chimera | Bi-allele | Homozygote |
|  | **HL64-17** | Mutation | -2 | +1/-5 | WT | WT | -26 | -14 | -5/-9 |
|  |  | Zygosity | Homozygote | Bi-allele | Wild | Wild | Homozygote | Homozygote | Bi-allele |
|  | **HL64-19** | Mutation | -1/-2 | +1 | +1 | +1 | -26 | -7/-14 | -5 |
|  |  | Zygosity | Bi-allele | Homozygote | Homozygote | Homozygote | Homozygote | Bi-allele | Homozygote |
|  | **HL64-512*** | Mutation | -1/-2 | +1 | +1 | +1 | -26 | -7/-14 | -5 |
|  |  | Zygosity | Bi-allele | Homozygote | Homozygote | Homozygote | Homozygote | Bi-allele | Homozygote |

-* Cas9 free plants.

**
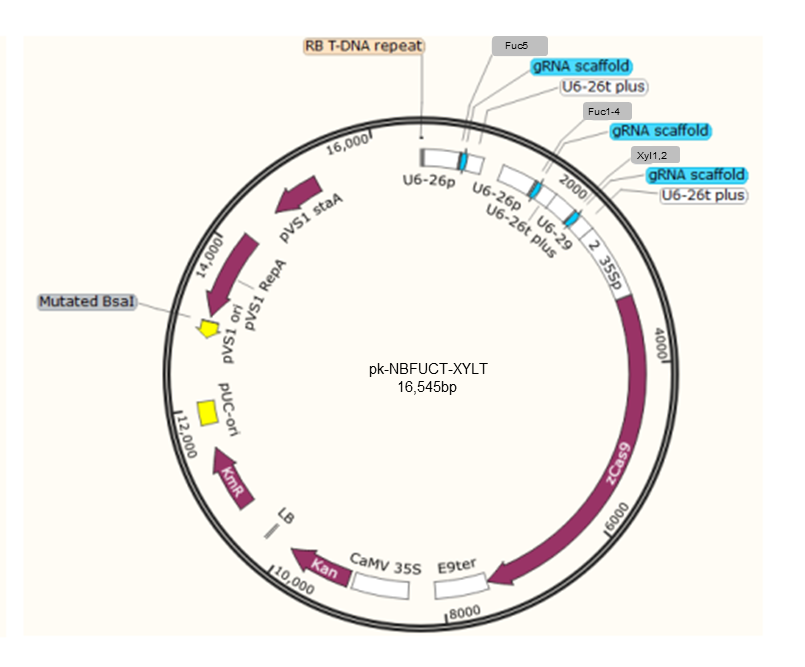
**

**Figure S1***. Nicotiana benthamiana* pk-NbFUCT-XYLT plasmid construct containing three sgRNAs targeting two NbXylT and five NbFucT genes.


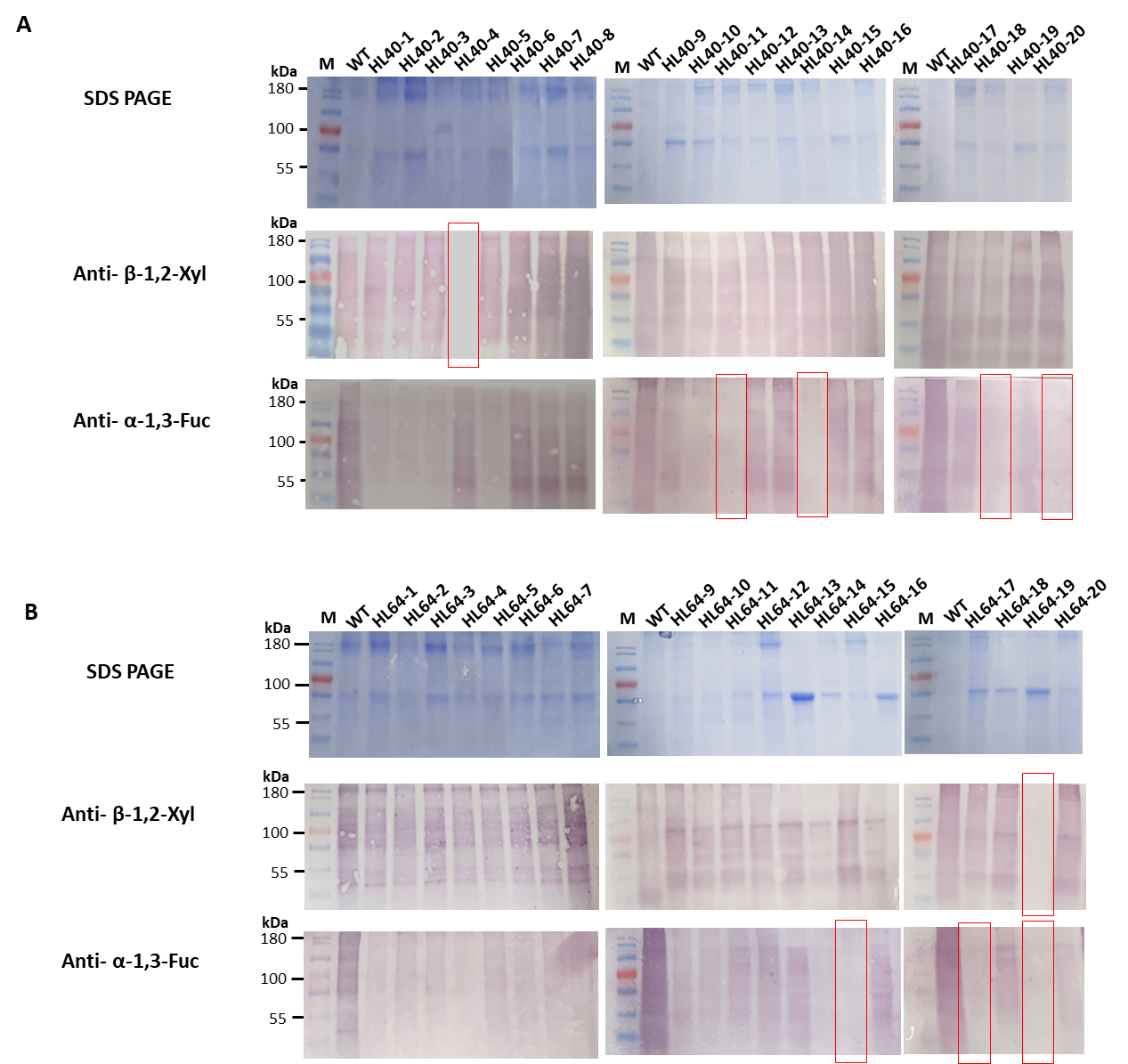


**Figure S2 .** Western blot analysis of the **(A)** HL40 and **(B)** HL64 F-KO and X-KO T_1_ transformants. Western blotting was performed using anti- β-1,2-xylose, and anti- α-1,3-fucose antibodies. The size of protein markers are indicated by kDa on the left side.


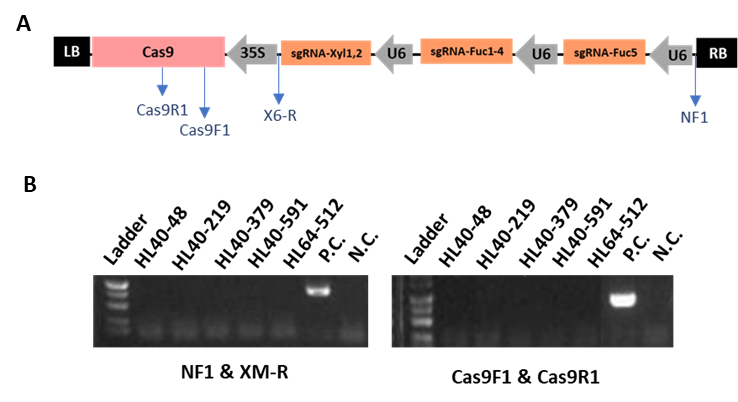


**Figure S3 .** PCR-based genotyping to check the absence of Cas9 gene in the T_1_ transformants **(A)** Schematic diagram of the Cas9-sgRNA construct and the position of primer set (Cas9 F1 x Cas9 R1). **(B)** Confirmation of the absence of Cas9 in T_1_ plants. Wild type *N. benthamiana* was used as negative control (N.C). The T_1_ line HL-64-19 was used as a positive control (P.C).
